# NextGen sequencing (NGS) panel for hereditary recurrent fevers: mutation spectrum, novel mutations, and evidence for re-classification of common variants based on analysis of >3000 cases from North America

**DOI:** 10.1186/1546-0096-13-S1-O55

**Published:** 2015-09-28

**Authors:** G Richard, C Lauricella, Z Xu, I Aksentijevich

**Affiliations:** 1 GeneDx, Gaithersburg, MD, USA; 2 NIH, NHGRI, Inflammatory Disease Section, Bethesda, USA

## Aim

Hereditary recurrent fevers (HRF) are genetically heterogeneous and often present a diagnostic challenge. To aid in molecular diagnosis, we developed and utilized a 7-gene NGS panel for HRF.

## Methods

The HRF panel includes *MEFV*, *MVK*, *NLRP3*, *TNFRSF1A*, *PSTPIP1, LPIN2* and *ELANE*, which are associated with familial Mediterranean fever (FMF), hyper-IgD syndrome, cryopyrin-associated periodic syndrome, tumor necrosis factor receptor-associated periodic syndrome (TRAPS), pyogenic sterile arthritis-pyoderma gangrenosum and acne syndrome, Majeed syndrome, and cyclic/severe congenital neutropenia, respectively. It utilizes a multiplex PCR approach, followed by NGS on HiSeq 2000/2500 instruments, and variant analysis using a custom-developed analysis pipeline.

## Results

Using this NGS panel, 3,248 individuals with suspected HRF were tested in our diagnostic laboratory. In this largely North American population, the majority of pathogenic/ likely pathogenic variants (PV/LPV) were identified in the *MEFV* gene (66.6%; n=187), followed by *MVK* (14.6%; n=41), *NLRP3* (8.9%; n=25), *TNFRSF1A* (3.6%; n=10), *PSTPIP1* (2.5%; n=7), *ELANE* (2.1%; n=6), and *LPIN2* (1.7%; n=5). Sixty percent of PV/LPV were recurrent mutations in *MEFV* (M680I, M694V, K695R, V726A), *MVK* (I268T and V377I) and *NLRP3* (R490K). The remainder were low-frequency or unique PV/LPV. Co-occurrence of pathogenic variants in 2 different genes was only observed in 2 families. Novel PV/LPV were identified in 5 genes, including *LPIN* associated with Majeed syndrome. Originally reported as pathogenic, the genetic contribution of several common variants to HRF remains unclear, including *MEFV*-E148Q and *TNFRSF1A*-P75L. In our cohort, both variants co-occurred with definite pathogenic mutations in *MEFV* or another fever-associated gene. While their minor allele frequency (MAF) in affected individuals was higher than in our exome sequencing controls (*MEFV*-E148Q: 2.8% vs. 1.9%; *TNFRSF1A*-P75L: 0.6% vs. 0.3%), numerous healthy individuals were homozygous for either variant. Newly available population data (1000 Genomes, ESP and ExAC) revealed a MAF for p.E148Q as high as 30% in Asians, and 10% for p.P75L in African individuals, including a large number of homozygotes (p.E148Q: 701/7768; p.P75L: 20/2775), which exceeds by far the prevalence of FMF and TRAPS in these populations.

## Conclusions

Our 7-gene NGS results represent the largest molecular-diagnostic dataset for HRF in the North American population, revealing mutation distribution and novel PV/LPV. We provide new evidence to reconsider the clinical significance of *MEFV*-E148Q and *TNFRSF1A*-P75L and propose these are population-specific polymorphisms that are unlikely to contribute to FMF or TRAPS. Our study underscores the utility of large datasets from diverse ethnic populations in clarifying the clinical significance of common HRF variants.

